# The influencing factors and generation path of digital health literacy among adolescents: evidence from 18 provinces in China

**DOI:** 10.3389/fpubh.2026.1700118

**Published:** 2026-01-29

**Authors:** Yi Wang, Qingwei Zeng, Mengyuan Chen

**Affiliations:** 1 School of Journalism and Communication, Guangzhou University, Guangzhou, China; 2 School of Education, Guangzhou University, Guangzhou, China

**Keywords:** adolescents, digital health, digital health literacy, digital media, intelligent media

## Abstract

**Introduction:**

This study explores the generation path and influencing factors of digital health literacy (DHL) among adolescents (aged 12–22), addressing gaps in the research by giving insufficient attention to underage youth and the lack of qualitative exploration.

**Methods:**

Adopting grounded theory, 74 semistructured online interviews were conducted with adolescents across 18 Chinese provinces, generating 190 thousand words of anonymized text. Through open, axial, and selective coding, 33 initial concepts, 14 core categories, and 6 main categories (literacy foundation, active exploration, passive arousal, positive effects, negative impact, and practical problems) were identified.

**Results:**

The results show that a literacy foundation (digital equipment/channels), active exploration (self-improvement/disease management), and passive arousal (media influence/environmental immersion) promote adolescent DHL, whereas negative impacts (health anxiety/information security) and practical problems (misinformation/supply shortages) hinder it. DHL benefits adolescents individually (fostering health actions, conceptual reconstruction, and habit formation) and socially (enabling digital feedback for health knowledge dissemination). This study also constructed an adolescent DHL generation path model and identified three paths, including the cognitive-environmental synergy path, action-technology driven path, and anxiety-action transformation path, collectively revealing the diverse formation mechanisms of adolescents’ DHL.

**Discussion:**

Practical suggestions for improving adolescents’ DHL were proposed. The results provided insights into enhancing adolescent DHL and support the optimization of adolescents’ health trajectory via digital tools.

## 1 Introduction

With the rapid development of digital media, intelligent media has increasingly become an important channel through which adolescents can obtain health information. It has also become an effective carrier for adolescents’ healthy development and health education. Digital health literacy (DHL) refers to an individual’s ability to obtain, understand, evaluate, and apply health information in a digital environment. It represents an individual’s ability to manage health in a digital environment. Previous studies of DHL among different groups, including college students and adults of other age groups, have reported that DHL directly affects their health behaviors and mental health (1–3). Additionally, these studies revealed significant variations in DHL by age (3).

As “digital natives,” adolescents (aged 12–22) are highly proficient in the use of digital tools. They are far more sensitive to the speed of information dissemination than adults are, yet their ability to identify false information may not yet be developed. In addition to providing guidance from parents or adults, adolescents gradually rely more on personal decisions and living environments in terms of health behaviors and habits (4, 5). However, current adolescents are faced with a series of problems, including false health information, an excess of general health information, health anxiety, and insufficient health equipment. These problems further increase health risks for adolescents in a digital society, becoming practical challenges for families, schools and educational management departments. This highlights the need for this population to develop sufficient DHL (6).

Additionally, major disease outbreaks are often accompanied by a surge of misinformation, which is exacerbated by social media networks (7). A study revealed that more than 95% of university students obtained information about the COVID-19 pandemic through new media platforms (8). However, a study revealed that 50.5% of the misinformation was disseminated through social media platforms (9). The COVID-19 pandemic accelerated digital health innovation and uptake and revealed the importance of digital health skills in accessing care and health information (2). Consequently, DHL has gradually become a hot topic of concern in the education sector and the health communication academic community.

Therefore, DHL helps adolescents establish health concepts, enhance their self-health efficacy, optimize health actions, and develop healthy habits, thereby improving their health quality. As adolescents reach adulthood, their ability to effectively obtain, process, and understand health information is crucial for making informed decisions and shaping their own health trajectories (10, 11). A substantial body of research indicates that compared with other populations, adolescents’ mental health is particularly susceptible to the impact of media communication. Moreover, the extant literature reveals significant gaps in the health literacy of this demographic, indicating a widespread inability to leverage health information for optimal health outcomes. The intervention of digital technology has further exacerbated these gaps (10). Therefore, in the era of rapid technological development, understanding the development path and influencing factors of digital literacy among adolescents is important.

However, adolescents’ DHL has been understudied, and the factors that drive its variation among adolescents require further exploration. As the popularity of digital health solutions increases, it is imperative to further understand the progression of DHL levels among adolescents. Previous studies have mostly used variable-centered quantitative methods to examine DHL correlates (12, 13), lacking qualitative analysis methods centered on individual and user experiences, which may blur the formation process and the heterogeneity of DHL among adolescents. This study addresses this knowledge gap by exploring the generation path and influencing factors of DHL among adolescents through grounded theory methods.

## 2 Literature review

### 2.1 Factors influencing DHL

The concept of DHL was first proposed by Norman and Skinner (14), who defined it as the ability to seek, discover, understand, and evaluate health information from electronic sources to address health issues. As research has progressed, this concept has gradually expanded to include not only the ability to acquire and understand information but also the ability to evaluate, apply, and create health information.

Research has indicated that good DHL is due mainly to a high level of digital skills (15). The application of computer-based knowledge and skills to specific organizational contexts, such as health, could also be important for a future professional career and should be developed over time with both education and expertise (16).

Research on the influencing factors of DHL focuses mainly on three aspects: individual characteristics, the social environment, and technological conditions. In terms of individual characteristics, research has shown that factors such as age, education level, self-awareness, and existing health status significantly affect DHL (17). Research suggests that individuals with higher DHL have higher self-rated health and mental health levels and a lower risk of developing chronic diseases (7). Researchers (18) proposed that health literacy can regulate an individual’s level of health by influencing their health behavior, which can help improve their self-health management ability and cultivate a healthy lifestyle. In contrast, a lack of health literacy can lead to a reduction in health behavior choices, a deterioration of health status, weakened self-management, and increased hospitalization rates (19). In addition, research has examined the impact of health information preferences on the DHL of college students (20). Research has shown that the level of social support received by middle-aged individuals can positively affect their level of digital health behavior and their health literacy has significant mediating and moderating effects on the relationship between the social support received and the digital health behavior adopted (21).

### 2.2 Measurement of DHL

In terms of measurement tools, from Norman and Skinner’s (14) earliest development of the Electronic Health Literacy Scale (eHEALS) to Van Der Vaart and Drossaert’s (22) DHL Instrument (DHLI), the measurement dimensions have gradually become richer, introducing interactive skills. The DHLI comprises 7 dimensions and 21 items and comprehensively assesses the level of DHL. Scholars have also developed specific scales for different populations, such as the Community Older adult DHL Assessment Scale (23), and the College Student DHL Scale (24), which provide support for measuring DHL.

Previous research has achieved some results, including defining the concept of DHL (25), developing measurement tools (26), and exploring influencing factors (27). However, research focuses mainly on the DHL of college students, clinical patients, and older adults (28, 29), with insufficient attention given to young people, especially underaged middle school students. Scale measurement or questionnaire survey methods are frequently used (30, 31), yet in-depth exploration using a combination of multiple methods is lacking. In addition, research on the development paths and internal mechanisms of DHL is lacking, especially the exploration of multiple paths. Therefore, this article proposes the following research questions:

Question 1: What are the paths for the formation of DHL among adolescents, and what are the similarities and differences between these paths?
Question 2: What factors promote or hinder the formation and development of DHL among adolescents?

## 3 Methods

### 3.1 Participants and sampling

The participants of this study were middle school and college students aged 12 to 22. Participants aged 19–22 years were college students who had recently transitioned to independent living but still faced adolescent-specific health challenges (e.g., mental health stress, sexual health education needs) and were in the early stages of establishing long-term digital health habits. Excluding this group would have omitted critical insights into how DHL develops during this transitional period. Adolescents at this stage are in a critical period of physical and mental development, and the formation of health literacy has a significant effect on their lifelong health. In this study, maximum variation sampling was used to select interviewees, ensuring diversity in geography, urban–rural status, age, and education and providing a representative sample.

The research team conducted in-depth interviews with the participants from July 2024 to January 2025. Geographical coverage includes 18 provinces in China, including Beijing, Shanghai, Guangdong, Shanxi, Henan, Hunan, Zhejiang, Fujian, and Jiangxi. Participants were identified through collaborative partnerships with local middle schools, colleges, and youth community centers in 18 provinces. School counselors and community workers initially screened potential participants, and our research team then approached eligible individuals via online meetings (for college students) or via guardians (for participants aged <18 years) to explain the study purpose and procedures. Inclusion criteria included (1) aged 12–22 years, (2) currently enrolled in middle school or college, (3) able to communicate in Mandarin or local dialects, and (4) willing to participate in online interviews. Exclusion criteria included (1) a history of cognitive or communication disorders and (2) no prior experience with digital health tools (e.g., health apps, online health information platforms). A total of 87 potential participants were invited, with 74 agreeing to participate (response rate = 85.1%) and 13 declining (decline rate = 14.9%). The main reasons for decline included time constraints and a lack of interest in digital health topics. The population segmentation was illustrated in Table 1.

**Table 1:** Participants characteristics.

Characteristic	Category	Number of participants	Percentage
Gender	Male	35	47.3%
	Female	39	52.7%
Age group	12–15 years	22	29.7%
	16–18 years	28	37.9%
	19–22 years	24	32.4%
Geographical distribution	Northeast China (Jilin Province, Liaoning Province)	7	9.5%
	North China (Hebei Province, Beijing, Tianjin)	11	14.9%
	East China (Shanghai, Anhui Province, Zhejiang Province, Fujian Province, Jiangxi Province)	22	29.7%
	Central China (Hubei Province, Hunan Province, Henan Province)	12	16.2%
	South China (Guangdong Province, Hainan Province)	10	13.5%
	West China (Shaanxi Province, Chongqing, Sichuan Province)	12	16.2%
Residence type	Urban	44	59.5%
	Rural	30	40.5%
Device ownership	Owned (including smartphones, computers, tablets, smart bracelets and other wearable devices)	67	90.5%
	Not owned	7	9.5%

### 3.2 Research procedure

A semistructured interview was conducted through online voice interviews. The interviews focus on individuals’ search, usage, sharing, and reception of digital health information, as well as their evaluation of their own DHL. The interview guide was developed in three stages. A preliminary guide was drafted based on a review of existing DHL literature (e.g., eHEALS, DHLI frameworks) and pilot interviews with 5 adolescents (not included in the final sample). The guide was revised to focus on open-ended questions about DHL experiences and generation paths. The final guide was reviewed by a qualitative research expert (not part of the team) to ensure clarity and alignment with our research aims. The full interview guide is provided in Appendix A. The average interview time was more than 40 min. Each interview was recorded and compiled into a written manuscript for archiving, resulting in approximately 190 thousand words of interview text that were anonymized. The interviewees are designated as P1 to P74.

The research team included three interviewers: one was a public health graduate student (with over 2 years of experience in adolescent health research), one was a practicing community health worker (with expertise in digital health promotion), and one was a middle school health educator (familiar with adolescent communication styles). All interviewers were proficient in Mandarin; for participants who preferred local dialects (*n* = 12), interviewers with native proficiency in the relevant dialect (e.g., Cantonese, Sichuanese) were assigned to ensure effective communication. To minimize bias, interviewers were instructed to avoid leading questions and to document their initial impressions of participants’ DHL levels in field notes—these notes were later discussed in team meetings to identify potential interpretive biases. The data analysts are PhDs and associate professors in the fields of health communication and adolescent education, with years of research experience in DHL education.

This study strictly adhered to research ethics standards and obtained approval from the ethics committee of the authors’ university before the start of the study. The approval reference number is [No. 2024–152]. For participants aged beyond 18 years old, a written informed consent was obtained via an encrypted online form (signed electronically using a secure digital signature tool), and a copy of the signed form was sent to participants via email. For participants aged under 18 years old, a guardian’s consent was first obtained via the same encrypted online form. Additionally, verbal assent was obtained from the underage participants themselves during the initial online meeting, which was audio-recorded (with guardian permission) and stored in a password-protected database as documentation.

### 3.3 Data analysis

This study adopted grounded theory to analyze in-depth interview data. Grounded theory is a qualitative research method that emphasizes the search for core concepts that reflect social phenomena from raw data. By summarizing, coding, and repeatedly comparing materials, we can discover the connections between different concepts and construct a theoretical framework that combines practice (32). This method is suitable for exploratory research, as it can summarize theoretical models from rich interview materials, which can aid in understanding the underlying mechanisms of adolescent DHL.

The grounded theory analysis of this study follows three stages, including open coding, axial coding, and selective coding. The data analysis process employing grounded theory typically unfolds through several iterative stages, each building upon the previous stage to construct theoretical insights inductively from empirical data. Two researchers used NVivo 11 software to extract concepts from the interview texts. After independent coding of 20% of the interview transcripts (a randomly selected subset) using Nvivo11, inter-coder consistency was assessed using Cohen’s Kappa coefficient. The calculated Cohen’s Kappa was 0.91. For the remaining transcripts, coding discrepancies between the two researchers were resolved through group discussions with a third senior researcher (with expertise in qualitative methods), until a consensus was reached. With respect to the inconsistent coding of the interview texts, the experts were asked to provide coding suggestions. In the stages of axial and selective coding, all four team members (two coders, one senior researcher, one public health expert) participated in weekly meetings to review subtheme relationships and core paths. Decisions about grouping subthemes into paths were made via majority vote (with all members required to document their rationales for dissenting opinions). Final consensus on the three paths was achieved after three rounds of discussion, with no unresolved disagreements.

In the open coding stage, two researchers independently coded interview transcripts line-by-line, using inductive labels (e.g., “Using smart sports wristbands”) to identify initial concepts. A total of 107 initial concepts were generated, which were then grouped into 33 subthemes (e.g., “Intelligent equipment”). In the axial coding stage, the team used a “coding paradigm” (33) to explore relationships between subthemes. For example, we identified “Physical ability improvement” as a “contextual condition” that influenced “Self-improvement” (a subtheme of the “Active seeking”). In the selective coding stage, we integrated core subthemes into six overarching main categories and further refined these categories to ensure they could account for the majority of participants’ experiences. For instance, we categorized “Media influence” and “Environmental immersion” under the dimension of “Passive arousal”; through this iterative integration and refinement, we ultimately established the theoretical framework of the “generation path of DHL for adolescents.”

To determine when to end data collection, we used a sequential approach. After each wave of interviews (10–15 participants per wave), the research team conducted a preliminary analysis to identify emerging themes. We continued recruitment until two consecutive waves (25 participants total) yielded no new themes or subthemes—a standard criterion for theme saturation in grounded theory (34). Additionally, no new relationship structures were generated between different categories. Therefore, in this study, the generation path and influencing factors model of adolescent DHL reached theoretical saturation.

## 4 Results

### 4.1 Open coding

By organizing, analyzing, comparing, and summarizing the collected in-depth interview data, and excluding irrelevant content, a total of 33 initial concepts were obtained. The same types were merged and aggregated into 14 initial categories, as shown in Table 2.

**Table 2:** Open coding results.

Core category	Initial concept	Original text of interviews
B1 Digital equipment	A1 Fitness tracker A2 Smartwatch A3 Intelligent equipment	P6: Sports wristbands are the most commonly used digital health equipment among classmates, and they also use digital smart devices such as smart scales and watches. P7: I first used a sports wristband because my sister, after using it herself, thought it was a good device for controlling heart rate and emotions, so she recommended it to me. P8: Our school has specialized health education courses, and teachers recommend reliable health apps and websites. We also have smart bracelets, body fat scales, and other devices at home.
B2 Digital channels	A4 Health APP A5 Health official account A6 Video Platform A7 Generative AI	P19: I usually pay attention to beauty and health apps, but my peers seem to be more interested in sports and fitness apps, such as Keep, Ant Forest, Quark Health Consultant, Mint, and so on. P20: I often watch bloggers sharing their healthy eating habits on Xiaohongshu (a short video app). P37: My health information is generally obtained from some well-known science bloggers on TikTok, BiliBili, Xiaohongshu, and microblogs (social networking platforms). P21: I usually use three apps: Mint Health (a health record app), Keep (an exercise teaching app), and Light Oxygen Medical Beauty (A beauty and health app). P39: I followed a WeChat public account called “Sports Science” and I also followed “Health Know All” (a WeChat official account). P40: I will send my personal information to Kimi (a generative artificial intelligence) and ask it to tailor a weight loss plan for me.
B3 Self-improvement	A8 Weight loss and fitness A9 Beauty and skincare A10 Health management A11 Nutrition improvement A12 Physical ability improvement	P3: I use Mint Health (a health record app) to record daily calorie intake; we can improve our dietary structure and achieve weight loss goals. P56: I want to improve my condition of acne and enlarged pores, so I will go online to learn related knowledge. P47: I am very concerned about how to use health monitoring devices, such as smart bracelets and smartwatches, to track physiological indicators, such as heart rate, steps, and sleep quality. P73: I pay special attention to the nutritional components of daily diet, such as protein intake and vitamin supplementation. P33: After I entered junior high school, the school’s requirements for physical fitness became higher. To meet the requirements of physical education teachers, I started to learn online how to improve my running speed. P5: I often read information about skin care and weight loss in the Little Red Book, and pay attention to some WeChat official accounts for mental health. P7: I mainly focus on fitness and muscle-building content, and often watch training tutorials on Bilibili.
B4 Disease management	A13 Intelligent monitoring A14 Online consultation A15 Intelligent early-warning	P24: I have hypoglycemia, and smart devices can help me monitor it and remind me to pay attention in time. P22: I had eczema before. Since medication could not cure it completely, I would search online for daily diet combinations to control the condition. P1: I suffer from asthma. Wearing an Apple smartwatch, I can monitor heart rate fluctuations at any time to warn of sudden asthma attacks, thereby better controlling the condition.
B5 Media influence	A16 News touches A17 Advertising persuasion A18 Blogger persuasion	P46: I read some reports stating that a lack of certain essential nutrients is harmful to the body, and since then, I have started paying attention to information in this regard. P7: I first saw an advertisement on WeChat saying that there was an app that could record sleep, analyze sleep quality based on your number of turns, sleep duration, snoring, etc., and even let you hear your own sleep talk when you wake up the next day, so I downloaded it to try. P16: There are authoritative bloggers who say that clothing color can affect mood, and I find it quite interesting. I will put this into practice.
B6 Environmental immersion	A19 Healthy community A20 Sharing from relatives and friends A21 Event triggered	P66: I often discuss health topics with netizens under the posts of some health bloggers. P5: My friend has been the most helpful to me in obtaining digital health information, and we often discuss training videos on video platforms with each other. P34: I have a classmate who has excellent grades and goes to bed at 2 a.m. every day to study. Unfortunately, he had heart problems and his family asked him to wear a wristband to school to monitor his heartbeat in real time. This also sparked my interest in this type of digital health device.
B7 Health actions	A22 Stress release A23 Skill learning A23 Risk aversion A24 Diet regulation A25 Fitness Action	P13: When I see myself under excessive stress on stress monitoring software, I tend to relax appropriately. P19: I can find many warm-up exercises and training techniques online, which are very helpful to me. P52: During exercise, the smartwatch will display my heart rate in real time. If it gets too high, I will stop exercising to ensure my safety. P64: I will adjust my diet plan for weight loss based on the advice provided by people in the peppermint health community and personalized services provided by the software. P67: I usually use the sports software Keep to engage in regular and periodic physical exercise, burn fat, lose weight, and tone my body.
B8 Conceptual reconstruction	A16 Eliminate mispronunciations A17 Reshaping concepts	P1: My parents think that “being able to eat is a blessing,” but after searching for relevant information online, I found that eating more is not necessarily better. P48: At first, I thought that the aesthetic of having “fair skin and slim figure” was the best, but after researching relevant information online, I realized that pursuing “beauty” on the basis of health is important.
B9 Habit formation	A18 Exercise supervision A19 Video follow-up practice	P62: The wristband can record my daily exercise amount in real time. Sometimes, due to studying or laziness, there may be significant differences in the data. The wristband also reminds me and urges me to keep exercising. P64: I practiced Baduanjin (a traditional Chinese fitness exercise) on Xiaohongshu (a short video platform), which made me develop a habit of fragmented exercise.
B10 Digital feedback	A20 Information forwarding A21 Voicing opinions online	P66: When I come across popular science articles about common diseases of middle-aged and older people, I will forward them to my elders to help them enhance their understanding of health science. P3: During COVID-19, I found that some people did not know the correct way to wear the N95 mask when I viewed the Little Red Book, so I would leave a message on their posts, which was later forwarded by many netizens.
B11 Health anxiety	A22 Disease anxiety A23 Health indicator anxiety A24 Equipment dependency	P5: I often receive articles about diseases on social media, which sometimes causes unnecessary anxiety. P38: There is too much information on the internet, some of which is overly one-sided and exaggerated, which can cause anxiety for me. P46: I have found that an excessive focus on physical data can lead to mental problems. Sometimes I forget to bring my sports wristband, and it feels like there’s no exercise without a record.
B12 Information security	A25 Privacy risk A26 Safety risk A27 Information confusion	P56: The current data flow is too fast, and the operators and information flow behind it are unknown. Everything is in a ‘black box’, and there is a great risk of privacy leakage. P61: Currently, many criminals judge our behavior based on the public health information on social media, such as exercise steps and the “Energy of Green Forests” (a function of a certain payment software that earns points through environmentally friendly travel behavior) information. I am very concerned about this, so I do not dare to activate these functions. P9: No one wants their information to be known by strangers, but I do not know how to ensure my information security.
B13 Attitude toward information	A28 Misleading information A29 Information questioning A30 False denunciation	P36: Once, I noticed that my urine was red. All the results from my online search were related to cancer, which scared me so much that my legs went weak. Later, I went to the hospital for a B-ultrasound, only to determine it was because I had eaten dragon fruit the night before. P37: Currently, there are many online self-media outlets run by people who are not doctors and have no qualifications. They edit some comments from netizens and spread them as medical knowledge, which is very misleading. P18: Some bloggers may engage in false advertising for the purpose of promoting products or advertising, but in fact, this product is truly ineffective. They are just trying to make money and mislead more people into buying this product. P3: I will cross-validate health information online to see if the claims from different sources are consistent, and consult some professional medical websites for content.
B14 Shortage of supply	A30 Platform blocking A31 Informational deficiencies A32 Lack of equipment A33 Scarcity of digital tools	P16: Health information about menstruation is easily blocked by public media platforms. P5: The health content related to the popularization and prevention of diseases based on the sex of men and women is basically not searchable. P59: I did not have my own electronic devices in high school, so I could not search and receive health information. P60: I think people tend to prefer using interactive and gamified health tools, but currently there is a lack of them. P1: Our network here is not very stable, and mobile phones are only available in high school. We mainly learn about health knowledge through school health education classes and guidance from family members.

### 4.2 Axial coding (associative coding)

The purpose of axial coding is to compare and analyze the initial concepts and basic categories obtained from the previous open coding step, attempt to find potential connections between each independent basic category, and then summarize and name them to form the main category. We further refined, integrated, and classified the 14 basic categories obtained from open coding, sorted out the logical relationships between the basic categories, and ultimately formed 6 main categories, namely, literacy base, active exploration, passive arousal, positive effects, negative impacts, and practical problems, as shown in Table 3.

**Table 3:** Axial coding results.

Main category	Core category	Initial concept
Literacy foundation	B1 Digital equipment	A1 Fitness tracker A2 Smartwatch A3 Intelligent equipment
	B2 Digital channels	A4 Health APP A5 Health official account A6 Video platform A7 Generative AI
Active seeking	B3 Self-improvement	A8 Weight loss and fitness A9 Beauty and skincare A10 Health management A11 Nutrition improvement A12 Physical ability improvement
	B4 Disease Management	A13 Intelligent monitoring A14 Online consultation A15 Intelligent early warning
Passive arousal	B5 Media influence	A16 News touches A17 Advertising persuasion A18 Blogger persuasion
	B6 Environmental immersion	A19 Healthy community A20 Sharing from relatives and friends A21 Event triggered
Health promotion	B7 Health actions	A22 Stress release A23 Skill learning A23 Risk aversion A24 Diet regulation A25 Fitness Action
	B8 Conceptual reconstruction	A16 Eliminate mispronunciations A17 Reshaping concepts
	B9 Habit formation	A18 Exercise supervision A19 Video follow-up practice
	B10 Digital feedback	A20 Information forwarding A21 Voicing opinions online
Negative impact	B11 Health anxiety	A22 Disease anxiety A23 Health indicator anxiety A24 Equipment dependency
	B12 Information security	A25 Privacy risk A26 Safety risk A27 Information confusion
Practical problem	B13 Attitude toward information	A28 Misleading information A29 Information questioning A30 False denunciation
	B14 Shortage of supply	A30 Platform blocking A31 Informational deficiencies A32 Lack of equipment A33 Scarcity of digital tools

### 4.3 Selective coding and theoretical model

Through open coding and associative coding, this study repeatedly examines and analyzes the relationships between the main categories, ultimately establishing the “generation path of DHL for adolescents,” and connecting the core category with other categories through the “storyline” of these phenomena.

**Figure 1:**
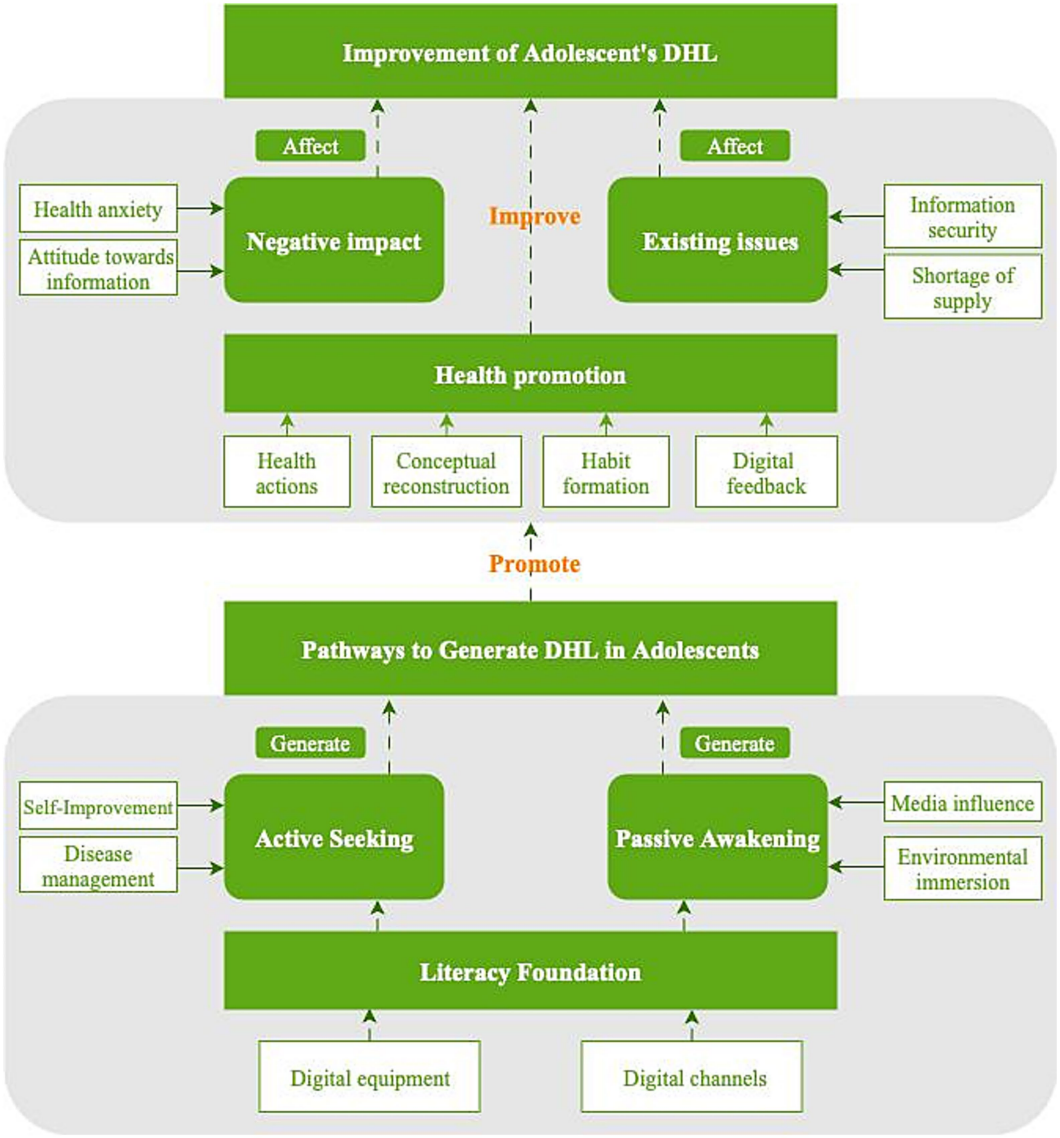
DHL generation path model for adolescents.

Using the software and hardware of *literacy foundation*, teenagers’ DHL begins to develop under the combined effects of *self-improvement*, *disease management*, *media influence*, and *environmental immersion*, among other factors, and has positive effects on teenagers (such as *health actions*, *concept reconstruction*, and *habit formation*). However, this formation process involves some potential impacts (such as *health anxiety* and *information attitudes*) and practical problems (such as *information security* and *supply shortages*). On this basis, a “DHL generation path model for adolescents” was constructed in this study (see Figure 1).

The results revealed that the *literacy foundation*, *active exploration*, *and passive arousal* constitute the antecedent variables for promoting DHL among adolescents, whereas *negative impact* and *practical problems* are influencing factors that hinder DHL among adolescents.

#### 4.3.1 Path 1: cognitive-environmental synergy path

Among the 74 participants, 46 (62.2%) of the participants’ DHL formation paths belong to this category. This path emphasizes the complementary role of cognitive reconstruction and environmental support. Conceptual reconstruction (e.g., cognitive changes regarding health information) and disease management capabilities (e.g., using digital devices to monitor health indicators) serve as the core drivers, while moderate health anxiety (e.g., concerns about diseases) and positive environmental influence (e.g., health-related discussions on social media or among relatives and friends) provide external impetus. For instance, Participant P1, who wore a smartwatch to monitor asthma conditions, gradually developed a scientific health management concept by combining this practice with health discussions in the family health community (environmental influence).

“*I have asthma. After wearing an Apple smartwatch, I can monitor heart rate fluctuations at any time to warn of sudden asthma attacks, thereby better controlling my condition. My family also shares articles about asthma management in our family group, which has given me a more comprehensive understanding of my condition.*” (P1)

This path indicates that the improvement of DHL cannot be achieved in isolation, but must be firmly based on cognitive transformation—specifically, individuals need to update their original perceptions of health information (e.g., shifting from passive acceptance of fragmented health content to active identification of evidence-based information) and establish a scientific understanding of digital health tools (e.g., recognizing the value of smart devices in real-time health monitoring), while also relying on the social environment for solid support, such as health-related discussions among family and friends, or reliable health information sharing on social media platforms, which provide continuous external impetus for the consolidation of cognitive changes.

#### 4.3.2 Path 2: action-technology driven path

Out of 74 cases, 19 (25.7%) participants’ DHL formation paths belong to this category. This path highlights the formation mode of DHL empowered by technical tools. The combination of health actions (e.g., regular use of fitness-related apps) and digital devices (e.g., smart bracelets) serves as a necessary condition, while information security awareness (e.g., attention to privacy risks) and disease management capabilities (e.g., adjusting health plans based on data) ensure the sustainability of such behaviors. For example, Participant P64 developed a dietary plan through the Bohe Health app and monitored heart rate via a smart bracelet; however, due to concerns about data leakage (information security), he/she only chose trusted platforms to obtain health information.

“*I adjust my dietary plan for weight loss based on the advice provided by members of the Mint Health community and the app’s personalized services. Meanwhile, I also pay close attention to information security and only access health information on formal, qualified platforms, as I do not want my health data to be shared arbitrarily.*” (P64)

This path clearly reflects that in the context of DHL development, tool usage and behavior reinforcement form a reciprocal promotional relationship: the effective application of digital health tools (e.g., fitness apps, smart bracelets) provides practical support for individuals to carry out standardized health behaviors, while the positive outcomes of these behaviors (e.g., improved physical indicators, simplified health management) further strengthen their willingness to continue using such tools. Specifically, the sustained practice of implementation and optimization of health behaviors, in turn, enhances the depth of tool application and the recognition of tool value, jointly facilitating the improvement of individuals’ DHL.

#### 4.3.3 Path 3: anxiety-action transformation path

Among the 74 participants, 9 (12.1%) of the participants’ DHL formation paths belong to this category. This path focuses on the positive transformation of emotional motivation. Health anxiety (e.g., concerns about obesity or chronic diseases), supported by digital devices (e.g., intelligent follow-along exercise systems), is transformed into specific health actions (e.g., daily fitness check-ins), while disease management capabilities (e.g., optimizing exercise intensity using data) ensure the effectiveness of these actions. For instance, participant P46 experienced anxiety due to excessive focus on weight data, but later gradually developed scientific fitness habits through the personalized exercise programs provided by a smart bracelet:

“*Excessive focus on physical data once caused problems with my mindset. Sometimes, if I forgot to bring my fitness bracelet, I would feel like I had not exercised without recording it. Later, I learned to make use of this anxiety, turning it into motivation to keep exercising. With the personalized advice from the bracelet, I can now arrange my exercise plans more scientifically.*” (P46)

This path further reveals the dynamic “emotion-action” transformation mechanism in the context of DHL development: it specifically demonstrates that health anxiety—an emotional state often triggered by concerns about physical conditions (such as weight gain or chronic disease risks)—does not have to lead to negative outcomes like excessive worry or inaction, but can instead be channeled into positive health behaviors (e.g., regular exercise, standardized diet management) with the support of technical tools, as these tools (such as smart bracelets with personalized guidance or fitness apps with progress tracking) provide tangible means to translate abstract anxious feelings into actionable, goal-oriented health practices.

## 5 Discussion

### 5.1 Factors promoting adolescents’ DHL

Based on the interview texts analyzed via grounded theory and the examination of pathways underlying adolescents’ DHL development, a set of positive factors associated with adolescents’ DHL formation was identified, primarily including *conceptual reconstruction*, *health actions*, *environmental immersion*, and *digital equipment*.

In the “cognition-environment synergy” path and “action-technology driven” path, *conceptual reconstruction*—as the cognitive foundation—was recognized as a key factor that correlates with adolescents’ DHL. This process involves adolescents proactively utilizing digital health information to reflect on and correct their existing (even erroneous) health perceptions, which in turn is linked to the establishment of evidence-based health cognition (35). For instance, one participant noted, “*I realized some of my unhealthy behaviors or symptoms of illness by watching health-related short videos”* (P26). Another participant reported that online information altered their understanding of sports injuries, which was followed by their “*deliberately adjust my usual sports habits—like avoiding the common issue of knee damage from playing soccer*” (P12). Such cognitive shifts provide supportive conditions for the development of DHL.

Across the “cognition-environment synergy” path, “action-technology driven” path, and “anxiety-behavior transformation” path, health-related behavior provides practical scenarios and social support that are relevant to DHL development (36). Adolescents are not passive recipients of health information but active practitioners of health management; this proactive engagement facilitates the translation of health information into health competence (a process that aligns with DHL improvement). For example, one participant stated, “*My classmates and I are quite interested in sports. They are especially into small daily health exercises shared on the platform—like last year, everyone was into practicing Baduanjin (a type of health exercise)”* (P42). This translation of online knowledge into offline action, coupled with dynamic adjustments based on personal health conditions (e.g., *disease management*), represents a critical link in the internalization of health knowledge into practical competence—an internalization process that is associated with DHL progression from cognition to action. Thus, health-related behavior—shaped through practice—functions as a key bridge that connects DHL from cognition to action.

In the “cognition-environment synergy” path, *environmental immersion* acts as a social catalyst that is linked to DHL development; a positive social environment shows a significant association with DHL formation (37). As developing individuals, adolescents’ health perceptions and behavioral patterns are profoundly influenced by social environments, including family health values, school health education, community health resources, and the media information environment. On one hand, *environmental immersion* may stem from health behavior modeling by family members and peers. When adolescents observe that “*classmates around me use smart devices (Apple Watches) to manage health, which seems novel and useful*” (P57), such peer influence and community atmosphere stimulate interests in imitation and participation that correlate with accelerated adoption of digital health practices. On the other hand, *environmental immersion* may also originate from media reports and short-video topics. One participant recalled, “*In high school, I accidentally came across a Douyin (a short video platform) video on simple weight-loss exercises. The content was engaging, so I started following health-related short videos afterward*” (P51).

In both the “action-technology driven” pathway and “anxiety-behavior transformation” path, *digital equipment*—frequently mentioned in interviews with urban adolescents—serves as “digital partners” for adolescents to explore health and understand themselves. Beyond basic functions like step-counting or timekeeping, these devices subtly contribute to the building of future-oriented DHL, which is the awareness and ability to proactively use digital technology for personal health management. Firstly, *digital equipment* encourages positive health behaviors and enables evidence-based interventions through real-time feedback and personalized reminders—behaviors and interventions that are associated with DHL improvement. One participant explained, “*It not only allows me to anticipate my health status in advance but also monitors my sleep quality, constantly alerting me to my physical condition*” (P55). Secondly, *digital equipment* helps adolescents build healthy communities, which enhance motivation for sustained health behaviors through social interaction. As one participant noted, “*I joined a community running group and often participate in the running rankings on digital platforms*” (P49). Additionally, participants highlighted that digital devices support accident prevention, “*The smartwatch has a feature—if you fall and do not get up within a certain number of seconds, it automatically calls emergency contacts. This function is quite practical*” (P72).

### 5.2 Factors hindering adolescents’ DHL

The results revealed that four key factors are associated with hindered formation of DHL among adolescents. First, health anxiety shows a clear dual association with the formation of DHL. Research has shown that moderate *health anxiety* can correlate with DHL, as it is linked to adolescents’ active seeking of health information. However, severe levels of health anxiety may be associated with psychological disorders, and excessive health anxiety may become a hindrance, resulting in increased psychological burden and even unnecessary panic. The experience of interviewee P5 illustrates this issue well: “*When using social media, they often push me about some illnesses, and sometimes they identify with them, causing anxiety*.” This discovery goes beyond the traditional negative understanding of health anxiety and reveals its potentially positive effects under moderate conditions, providing a new perspective for understanding the complexity of health anxiety.

The accuracy of digital health information is the second important factor that correlates with the formation of DHL among adolescents. Research has shown that the widespread dissemination of misinformation on the internet (*misleading information*) and the widespread questioning of the credibility of health data (*information questioning*) create challenges for adolescents to access and apply health information. The experience shared by interviewee P36 vividly reflects this issue: “*Once when I went to the bathroom, I found that my urine was red, and the results of online searches were all related to cancer, which scared me so much that my legs went weak. Later, I went to the hospital specifically for an ultrasound and determined that it was because I had eaten dragon fruit the night before*.” P16 believes that the credibility of health data is questionable (i.e., ‘*I have bought several different brands of fitness bracelets, but found that when I use them for the same exercise, the differences in health data are quite large, making me unsure which one to believe*’). This unnecessary panic caused by inaccurate information not only relates to the mental health of adolescents but may also be associated with a general lack of trust in health information. These findings indicate that teenagers’ attention to the accuracy of information shows a significant association with the formation of literacy.

*Information security* concerns constitute the third type of factor that is associated with hindrances to DHL. Research has revealed that teenagers have significant concerns about the security of digital health information, which is manifested mainly in three aspects (i.e., *privacy risks*, *safety risks*, *and information confusion*). Previous studies have shown that perceived risk has a direct effect on the behavioral intention to use information systems (5, 16). P61’s opinion represents the concern of many teenagers: “*Now, many lawbreakers know your whereabouts according to your health information published on social media, such as the number of WeChat sports steps and the ‘energy’ of Alipay Ant Forest. I am worried about this, so I do not dare open these functions*.” This safety concern exacerbates negative experiences of using digital health tools among teenagers, which directly correlates with their willingness to use digital health tools and thus is associated with obstacles to improving DHL.

*Shortage of supply* is the fourth type of factor that correlates with hindrances to DHL. Research has shown that there are serious issues with the homogenization of content, excessive blocking of sensitive information, and unmet specific needs (such as gender knowledge and mental health) in the current supply of health information for adolescents. In Davis’ (1989, 33) technology acceptance model, users’ attitudes and behavioral intentions toward the use of new technologies are influenced by both perceived usefulness and perceived ease of use. Among them, perceived usefulness refers to the degree to which users perceive the benefits of using technology. An uneven supply of information greatly weakens the perceived usefulness of digital technology for adolescent users. Multiple interviewees reported that: “H*ealth information related to gynecology, such as menstruation, is easily blocked by platforms, and health content related to the popularization and prevention of diseases on the basis of male and female sex characteristics is essentially not searchable*.” In addition, the lack of digital health infrastructure in school environments also limits the channels through which teenagers access digital health information. The imbalance and insufficient information supply make it difficult for teenagers to obtain comprehensive and scientific health knowledge, reducing their willingness to use technology and thereby affecting the comprehensive development of their DHL.

### 5.3 The formation paths of adolescents’ DHL

Based on a comparative analysis and relational examination of the promoting and hindering factors in DHL formation, this study identified three primary pathways through which adolescents develop DHL: the “cognition-environment synergy” pathway, the “action-technology driven” pathway, and the “anxiety-action transformation” pathway. The Health Action Process Approach (HAPA) (38) model posits that health behaviors should be viewed as a dynamic process spanning from motivation to maintenance, involving an analysis of individuals’ cognition, planning, and actions across different stages. This theory provides insights for analyzing the formation pathways of adolescents’ DHL in this study. To further clarify the characteristics and relationships of the three pathways, their key features, process traits, high-frequency concepts, and other relevant information are summarized in Table 4.

**Table 4:** Description of DHL formation paths.

Path type	Characteristic of the process	High-frequency concepts	Key features
Cognition-Environment Synergy	Perceptual reconstruction that is associated with environmental immersion (including interpersonal and media influences), which in turn correlates with health-related behavior.	Environmental immersion Conceptual reconstruction Health actions	Emphasizes the complementary effect of health perceptions, health behaviors, and environmental support
Action-Technology Driven	Increased attention to health information that correlates with the use of digital devices, with lifestyle and health behaviors adjusted based on data.	Digital equipment Information security Conceptual reconstruction Health actions	Digital devices serve as external factors that correlate with health-related behavior
Anxiety-Behavior Transformation	Health anxiety arising from physical conditions or disease management, which is linked to health management or health-related behavior via digital device monitoring.	Disease management Digital equipment Health anxiety Health actions	Internal health factors trigger processes that are associated with digital health management and digital health-related behavior

The cognitive-environmental synergy path emphasizes the complementary effect of cognitive reconstruction and environmental support. Within this pathway, adolescents discard erroneous health perceptions, reshape evidence-based health cognition, and integrate environmental immersion—such as health discussions from social media or relatives/friends—along with appropriate attention to their health status. This process enables them to develop the ability to scientifically understand and apply health information, which in turn drives health-related behavior. For example, Participant P1 stated: “*My parents believe that ‘being able to eat well is a blessing,’ but after researching relevant information online, I realized that eating more is not necessarily better. Later, I adjusted my eating habits*.” This pathway aligns with the “cognition-behavior” theoretical model, indicating that improving DHL among adolescents requires cognitive enhancement as the foundation and social environment as the support.

The action-technology driven path highlights a health practice orientation empowered by technological tools. The combination of health-related behavior (e.g., regular use of fitness apps) and digital devices (e.g., smart bracelets) is a prerequisite, while awareness of information security (e.g., concerns about privacy risks) and health management capabilities (e.g., adjusting health plans based on data) ensure the sustainability of such behaviors. For instance, Participant P64 used a Health app to create a dietary plan and a smart bracelet to monitor heart rate. However, due to concerns about data leakage (information security), they only accessed information from trusted platforms. Overall, this pathway reflects the practical logic that tool usage and behavioral reinforcement mutually promote each other, providing technological impetus for improving adolescents’ DHL.

The anxiety-action transformation path emphasizes that internal health factors trigger digital health management and digital health-related behavior. The Health Belief Model (HBM) (39) notes that perceived disease threats can motivate individuals to adopt health behaviors. In this pathway, adolescents translate concerns about their health status (e.g., anxiety about obesity or chronic diseases) into specific health-related behaviors with the support of digital devices, and ensure the effectiveness of these behaviors through disease management capabilities. Participant P46, who wore a smartwatch to monitor asthma symptoms, gradually developed a scientific health management mindset by combining this monitoring with discussions in a family health group: “*I have asthma. After wearing an Apple Watch, I can monitor heart rate fluctuations at any time to alert me to asthma attacks, which helps me better control my condition. My family also shares articles about asthma management in our family group, which gives me a more comprehensive understanding of my illness*” (P46). Health-related behavior triggered by internal health issues fosters a stronger sense of identity and urgency among adolescents, making this a typical internally driven pathway that provides self-motivation for improving DHL.

Additionally, it is particularly worth noting that disease management is not a marginal or optional factor but a universal component present in all three paths. In the cognitive-environmental path, it works with conceptual reconstruction to be relevant to DHL literacy development; in the action-technology path, it ensures the sustainability of tool-supported health behaviors; and in the anxiety-action path, it guarantees that anxiety-derived health actions achieve practical health effects. This cross-path consistency clearly demonstrates the core role of disease management in shaping adolescents’ DHL: for adolescents, the ability to use digital tools to monitor health indicators, adjust health plans, and respond to potential health risks (i.e., disease management capacity) is not only a direct manifestation of DHL but also a foundational prerequisite for integrating cognitive, behavioral, and emotional factors to promote the overall improvement of literacy. Furthermore, health anxiety appears in Path 1 and Path 3, health actions emerge in Path 2 and Path 3, and digital devices are present in Path 2 and Path 3. This indicates that these factors play important roles in different path combinations.

### 5.4 Limitations and future directions

This study has several limitations. The sample size of 74 adolescents, despite covering 18 provinces, is relatively small and may not fully reflect the regional differences and age stratification characteristics of Chinese adolescents. The imbalance in digital infrastructure and educational resources between urban and rural areas may not be adequately represented. The research relies on qualitative data from semistructured interviews, which carry risks of recall bias and social desirability bias and lack objective behavioral measurements, leading to insufficient exploration of the practical application depth of DHL. Additionally, the rapid changes in the digital health environment may make the data collected in 2024 unable to cover the impact of new technologies and policies, and the dynamic interactions between various influencing factors have not been analyzed in depth.

Future research can adopt mixed methods, expand the sample size to improve representativeness, and focus on the particularities of groups such as rural and ethnic minority adolescents. It can track the evolution of literacy through longitudinal and experimental designs, verify the effectiveness of intervention measures, explore the impact of new technologies such as generative AI on adolescents’ information processing abilities, and construct a multisubject collaborative ecological framework. Furthermore, it is necessary to develop targeted measurement tools, design school or community intervention programs, and conduct in-depth research on the role of “digital feedback” and peer networks in health information dissemination to provide more precise strategic support for improving adolescents’ DHL.

## 6 Conclusion and suggestions

This study systematically investigates adolescent DHL using grounded theory, filling research gaps in underage youth-focused and qualitative DHL studies. It identifies key antecedents (literacy foundation, active exploration, and passive arousal) that drive adolescent DHL formation and barriers (negative impacts such as health anxiety and practical issues such as misinformation) that impede it, further constructing a DHL generation path model. The findings confirm DHL’s dual positive effects: individually, it promotes healthy actions, corrects misconceptions, and builds habits, while socially, it enables adolescents to spread health knowledge via digital feedback, aiding intergenerational communication and public health literacy. This study yields actionable implications for educators, guiding them to address gaps in adolescent DHL development and leveraging supportive factors to enhance adolescents’ ability to navigate digital health environments.

First, educators should prioritize strengthening the literacy foundation—a core antecedent of DHL—by integrating digital health tools and channels into teaching. Given that digital equipment (e.g., fitness trackers, smartwatches) and channels (e.g., reliable health apps, official accounts, generative AI) lay the groundwork for DHL, schools could collaborate with families to ensure equitable access to such resources, especially for adolescents in rural or under-resourced areas (to mitigate “supply shortages” identified in the study). Educators can design hands-on workshops to teach adolescents how to use evidence-based health apps (e.g., Mint health, Keep) or evaluate generative AI-generated health plans (e.g., weight loss regimens), equipping them with practical skills to engage with digital health tools safely and effectively.

Second, educators should adopt strategies to stimulate both the active exploration and passive arousal of DHL. To foster active exploration, curricula can incorporate topics aligned with adolescents’ self-improvement needs (e.g., weight management, skincare, physical fitness) and disease management (e.g., intelligent monitoring for chronic conditions such as asthma). Health education classes could include projects in which students track their physiological indicators via smart devices and adjust their lifestyles on the basis of data, reinforcing the link between DHL and health actions. To leverage passive arousal, educators can integrate media literacy into lessons, guiding adolescents to critically analyze health information from news, advertisements, and bloggers (addressing “media influence” and “environmental immersion”). This includes teaching skills to identify misleading content (e.g., exaggerated disease claims) and cross-validate information with professional medical sources, as exemplified by interviewees who verified online data through hospital consultations.

Third, educators must proactively address barriers to DHL to prevent negative impacts. To alleviate health anxiety, lessons can normalize moderate concern about health while emphasizing evidence-based decision-making—for instance, discussing cases where excessive focus on fitness tracker data led to anxiety and teaching strategies to balance data use with mental well-being. With respect to information security, educators should deliver targeted sessions on protecting privacy (e.g., avoiding oversharing exercise data on social media) and recognizing risks such as “black box” data flows, empowering adolescents to use digital health tools without compromising safety. Additionally, educators can advocate for a more inclusive health information supply (tackling “supply shortages”) by requesting that platforms reduce the blocking of gender-specific content (e.g., menstruation-related health information) and by collaborating with health organizations to develop age-appropriate, diverse digital health resources for classrooms.

Finally, educators should facilitate digital feedback to extend the social impact of DHL. Encouraging adolescents to share verified health knowledge with family or peers not only reinforces their own DHL but also promotes intergenerational health communication and public health literacy. Schools could organize “health info ambassadors” programs where students create and share short digital content (e.g., videos, infographics) on evidence-based health practices, turning DHL into a tool for community health improvement. In conclusion, this study provides a reference for empowering educators to act as facilitators of adolescent DHL—by building foundational skills, stimulating engagement, mitigating barriers, and fostering social impact—ultimately supporting adolescents in using digital tools to optimize their health trajectories.

## Data Availability

The raw data supporting the conclusions of this article will be made available by the authors, without undue reservation.
