# Supplementary material for: The influencing factors and generation path of digital health literacy among adolescents: evidence from 18 provinces in China
Source: Front Public Health. 2026 Jan 29;14:1700118. doi: 10.3389/fpubh.2026.1700118 (PMC12895680; doi:10.3389/fpubh.2026.1700118)
Supplement: Supplementary file 1 [file Supplementary_file_1.docx]

**Appendix A**

# Interview Questionnaire on Adolescents’ Digital Health Literacy (Semi-structured)

1. What aspects of digital health literacy do you usually pay attention to? (To explore the content characteristics of adolescents’ digital health literacy from a structural perspective)

Please talk about the digital health-related skills or knowledge you pay special attention to in daily life, such as using health monitoring devices, understanding health data analysis, online medical consultation, using sports apps, using health apps, etc. Are you interested in beauty, weight loss, mental health, dietary health, or a specific physical disease? In your opinion, which aspect are your familiar peers more interested in?

1. When did you start paying attention to digital health literacy? What is the fundamental reason and origin that led you to focus on it? (To mainly understand the formation path of adolescents' digital health literacy)

Please share the time point when you first became interested in digital health literacy, and what prompted you to start focusing on this field, such as personal health needs, social hot events, recommendations from family or friends, etc.

1. Through which media channels do you obtain digital health information? Where do you mainly get your digital health information from? In your view, through which media or channels do most adolescents currently obtain digital health information?

Describe the channels you often use to obtain digital health information, such as social media, professional health websites, apps, TV health programs, offline lectures, etc., and share the channels you think are commonly used by adolescents. Through which channels do your familiar peers usually obtain digital health information?

1. Among the people around you, who provides you with or recommends digital health information?

Talk about who (such as family members, teachers, classmates, friends, colleagues, doctors) has an important influence on your acquisition of digital health information in your life, and how they influence you. From the perspective of the social groups you are familiar with or interact with, which groups are more helpful to you in obtaining digital health information?

1. What is the most impressive digital health event to you? Which piece of digital health information has left a deep impression on you?

Share a digital health-related event or piece of information that you remember deeply, and explain why it left such a deep impression on you.

1. What kind of health help do you think digital intelligent devices have brought to you? (Please talk about the 2 most impressive things)

Based on your own experience or true feelings, talk about how digital intelligent devices such as smart bracelets, smart watches, and health monitoring apps have improved your health condition, such as improving sports efficiency, monitoring sleep quality, and providing early warning of health risks. If you don't have such devices yourself, you can talk about your peers you have observed.

1. In your opinion, what advantages does the digital health information provided by current media, society, or in reality have? What are the good aspects?

Based on your own experience or true feelings, talk about the advantages of current digital health information, such as large information volume, fast update speed, convenient access, personalized recommendations, etc., and point out the specific benefits.

1. In your opinion, how has the digital health information provided by current media, society, or in reality helped shape your health concepts and health behaviors?

Based on your own experience or true feelings, talk about how digital health information has changed your health concepts, such as paying more attention to prevention and data monitoring, and how these changes have guided you to adopt healthier behaviors.

1. In your opinion, what problems still exist in the digital health literacy provided by current society? For example, in terms of supply quantity, supply quality, authenticity, supply structure, supply sources, etc.

Based on your own experience or true feelings, talk about the problems existing in the field of current digital health literacy, such as information overload, uneven quality, prevalence of false information, insufficient coverage of specific groups, etc.

1. In your opinion, do adolescents currently pay attention to the privacy of their own digital health literacy? How do adolescent users ensure that their health data is not misused or leaked?
2. What are the current needs of adolescents for digital health literacy education? How to design educational content and forms that are more in line with the needs of adolescents?

Combined with your own feelings, talk about what content you currently need in terms of digital health but cannot obtain. That is, what is lacking in the content of digital health communication for adolescents in current society?

1. In the era of information explosion, how can adolescents effectively verify the authenticity and reliability of digital health information? Are the existing verification mechanisms sufficient? When facing a wide variety of health information, how can adolescents develop their critical thinking skills to avoid being misled or deceived?

Combined with your own feelings, talk about how you identify the authenticity of digital health information. Do you believe all the information you see, or only part of it? In your opinion, how should adolescents develop their ability to identify the authenticity of digital health information?

1. If we want to establish evaluation indicators for adolescents’ digital health literacy, what characteristics do you think should be included?

In your opinion, what internal characteristics and external behaviors do your classmates or friends with good digital health literacy usually have? For example, having a wide range of health knowledge, often using digital health devices, often reading health-related books, often participating in lectures or training, liking to make friends who love health and sports, often showing their health behaviors or health concepts on WeChat Moments, or investing a lot of money in purchasing digital health devices?

1. Do adolescents have the behavior of sharing and communicating digital health information? Which social media or community do you think is your favorite platform for health information?

Combined with your own experience, talk about whether your familiar classmates and friends share adolescent health information through social media. For example, sharing on WeChat Moments, discussing health behaviors or health topics together in a certain social group, or discussing a certain health topic on Zhihu or Bilibili communities.

Combined with your own feelings, talk about which social media or community is your favorite platform for health information.

1. From the perspective of communication, what measures do you think we can take to improve personal digital health literacy? (For example, optimizing digital health information, building digital health communication platforms, establishing digital health communication teams, designing digital health mini-programs, developing digital health apps)

Combined with your own feelings, talk about your suggestions, such as strengthening the health information review mechanism, developing easy-to-understand health education content, using big data and AI technology to push health information personalized, and creating health education platforms that combine online and offline.

1. What significance do you think the improvement of digital health literacy has for yourself? What are the long-term impacts of the improvement of digital health literacy on individual adolescents and society? How will digital health literacy affect adolescents’ lifestyles, health concepts and social behaviors?
2. From the perspective of digital communication, what strategies do you think we can take to improve adolescents’ digital health literacy?

Combined with your own feelings, what kind of help do you think digital intelligent technology can bring to the improvement of adolescents’ digital health literacy? In which aspects can we give play to the advantages of intelligent communication?
